# Membrane fractionation of hydrolysates of the water-soluble protein from tuna fish meal obtained by subcritical water and enzymatic treatments. Comparison of physical and chemical properties

**DOI:** 10.1186/s40643-025-00850-3

**Published:** 2025-03-06

**Authors:** Pedro Barea, Alba Ester Illera, Helena Candela, Rodrigo Melgosa, José Manuel Benito, Sagrario Beltrán, María Teresa Sanz

**Affiliations:** Department of Biotechnology and Food Science, University of Burgos, Plaza Misael Bañuelos s/n, 09001 Burgos, Spain

**Keywords:** Protein hydrolysis, Dead-end filtration (UF and NF), Protein and Free amino acids profile, Reducing and chelating capacities, Molecular weight distribution

## Abstract

Two different hydrolysates of the water-soluble protein (WSP) fraction from tuna fish meal were obtained by subcritical water-CO_2_ (subW-CO_2_) and Alcalase^®^ treatments. Hydrolysates showed different chemical composition regarding their free amino acid (FAA) profile and molecular weight distribution of the peptides generated. Consequently, different strategies were proposed by using a stirred dead-end filtration system equipped with flat sheet membranes. Two nanofiltration (NF) consecutive steps were proposed for fractionation of subW-CO_2_ hydrolysates with polyamide membranes (800–600 Da and 300–150 Da) yielding a first retentate where 79% of the protein fraction was retained, while 92% of FAA were retained in the second step with a purity index of 29%. Alcalase^®^ hydrolysates were fractionated by ultrafiltration (UF, 10 kDa) followed by NF (1–1.1 kDa) with polyethersulfone membranes obtaining a first retentate with more than 65% of protein fraction retention with a purity index of 50%. A relatively wide molecular weight distribution was observed in all streams, with high antioxidant and iron-chelating capacities in the retentate fractions.

**Graphical Abstract:**

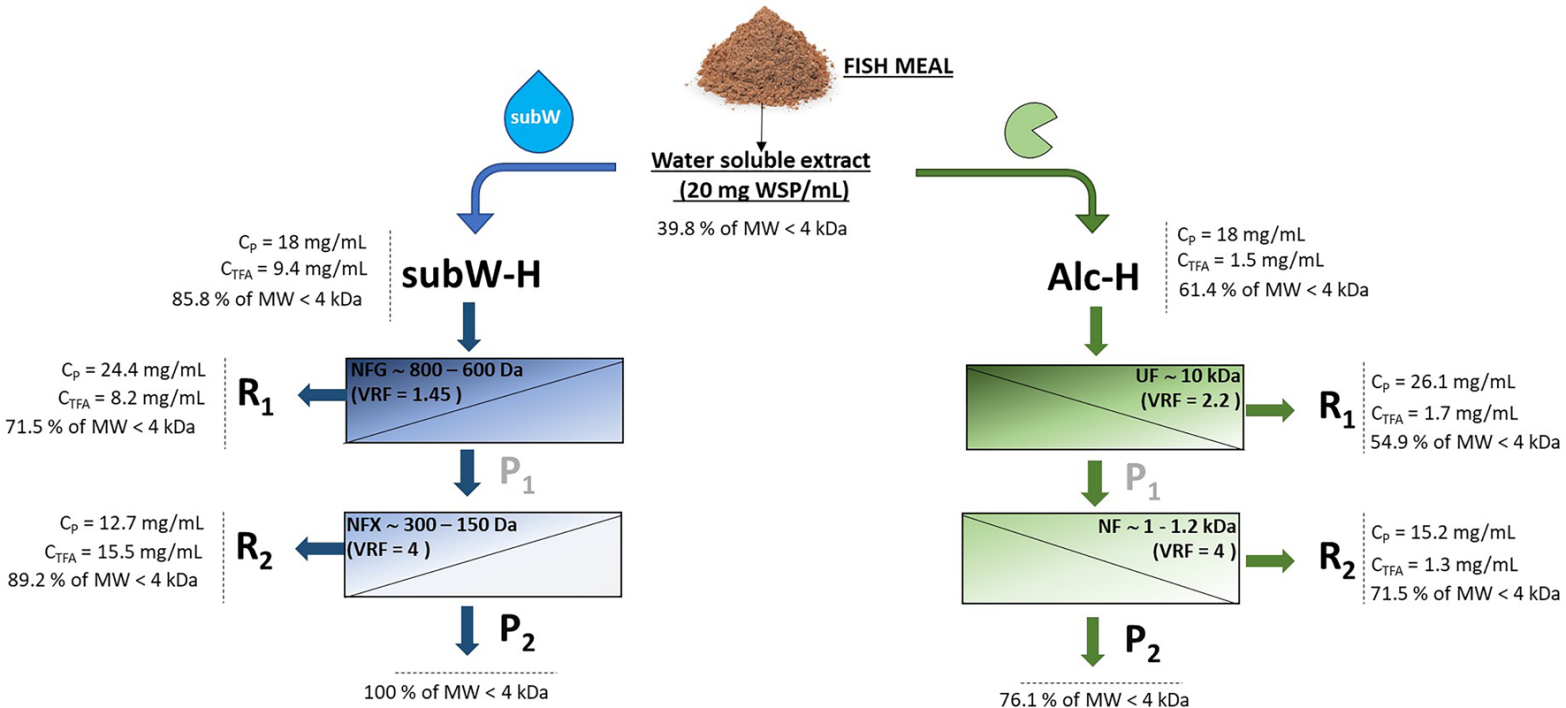

## Introduction

Fisheries generate various solid by-products, such as filleting waste or heads which are typically converted into fish meal and oil. Fish meal is an essential ingredient in aquaculture due to its high protein content and nutritional value. However, there is a need to explore better methods for upgrading fish meal by discovering new valuable products within the fish meal production chain. In this regard, fish protein hydrolysates are considered highly promising in the food, pharmaceutical, and cosmetic industries due to their excellent nutritional, functional, and biological activities (Bourseau et al. 2009; Barea et al. 2024). Fish protein hydrolysates consist of amino acid sequences, often between 2–20 residues, embedded within the natural protein structure. The production of fish protein hydrolysates emerges as a promising approach to add value to fish by-products, offering improved properties compared to the native protein.

This study focuses on the production and fractionation of protein hydrolysates derived from tuna fish meal. Previous research has explored the potential of water as an environmentally friendly extraction and hydrolysis agent for this material (Barea et al. 2023). In the present work, the initial step involved extracting the water-soluble protein (WSP) fraction from tuna fish meal through water extraction at 80 °C. Subsequently, this WSP underwent hydrolysis using subcritical water (subW) treatment by employing CO_2_ as a pressurization agent (subW-CO_2_). subW refers to water in its liquid state within the temperature range of 100 to 374 °C and pressures up to 22 MPa. Under these specific conditions, water exhibits unique properties. Notably, the increased concentration of H^+^ and OH^−^ ions in the aqueous medium enhances its catalytic activity, facilitating both acid- and base-like catalyst for hydrolysis reactions (Marcet et al. 2016). Additionally, the introduction of CO_2_ into the subW medium increases acidity through carbonic acid formation, further catalyzing WSP hydrolysis. Hydrolysates obtained by subW-CO_2_ treatment (from now on subW-H) were compared with those from conventional enzymatic hydrolysis with Alcalase^®^ (from now on Alc-H), showing different chemical composition in terms of the release of free amino acids (Barea et al. 2023).

In the literature, a relationship has been stablished between the physicochemical characteristics of peptides, such as their molar mass distribution and their biological activity. Specifically, fractions between 1 and 4 kDa are considered the most promising for nutritional and/or pharmaceutical uses (Saidi et al. 2014a). Hence, it is worthwhile to further investigate fractionation and concentration steps of the peptides generated during hydrolysis (Abejón et al. 2018).

Membrane separation processes have been proposed for the fractionation of peptides and other valuables biomolecules. Membrane technology offers a clean separation process that can be conducted under mild conditions with relatively low energy consumption. It also provides high flexibility in system design. Several studies have investigated the fractionation of marine hydrolysates, employing methods such as ultrafiltration (UF) or combined UF-nanofiltration (NF) processes to isolate fractions enriched in specific molecular weight (MW) peptides or free amino acids. These approaches aim to develop novel products with enhanced functional properties. However, the majority of existing studies have exclusively focused on the fractionation of hydrolysates obtained through enzymatic treatments with proteases, neglecting the exploration of more sustainable hydrolysis processes such as those subW-H (Picot et al. 2010; Saidi et al. 2013; Roslan et al. 2018; Abejón et al. 2018; Pezeshk et al. 2019; Chorhirankul et al. 2024).

The objective of this study was to investigate the integration of different hydrolysis treatments, applied to the WSP of tuna fish meal, along with membrane separation technology. Due to the significant release of free amino acids by subW-CO_2_ treatment, various membranes were initially tested using a synthetic mixture of the major amino acids released by subW-CO_2_ treatment. Based on these results, a two-step consecutive NF membrane process was studied for the fractionation of subW-H and a two-step UF (1st step) and NF (2nd step) membrane process for Alc-H. The different fractions generated during the membrane processes were characterized in terms of their chemical composition, focusing on protein content and free amino acids, hydrolysate size distribution and antioxidant activity.

## Experimental section

### Raw material

Tuna fish meal (*Thunnus sp.*) was used as the raw material in this study. It was kindly provided by Sarval Bio-Industries Noroeste, S.A.U. (A Coruña, Spain). The chemical composition of fish meal has been previously reported by Barea et al. (Barea et al. 2023).

The protein content was 51 ± 2% (w/w) as determined by Barea et al. considering as N-factor a value of 5.0 previously established and the N-elemental content (Barea et al. 2023).

### Water soluble protein extraction

The WSP was extracted by applying the optimal extraction conditions previously determined by Barea et al. (Barea et al. 2023). Specifically, 16 g fish meal per 100 mL of distilled water were put in contact at 80 ºC for 30 min. Subsequently, the water soluble extract was separated from the solid, the non-soluble water fraction, by centrifugation at 5000 rpm for 15 min. The WSP was then subjected to hydrolysis by subW-CO_2_ and enzymatic treatments.

### Hydrolysis by subcritical water-CO_2_ treatment

200 mL of WSP from fish meal were charged in a batch high pressure reactor of 0.5 L capacity. The reactor was covered by a ceramic heating jacket (230 V, 4000 W, ø 95 mm, 160 mm height) to achieve the selected working temperature. A Pt100 sensor placed inside the reactor was connected to a PID system to control and register the temperature during the hydrolysis process. The temperature for the subW-CO_2_ process was selected as 180 ºC and the working pressure was set at 50 bar by using CO_2_ as pressurization agent. Temperature and pressure were selected based on previous work by Barea et al., where subW hydrolysis was studied within a temperature range of 140–180 ºC, showing a positive effect of temperature in the hydrolysis process (Barea et al. 2023). The use of CO_2_ as pressurization agent enhanced the production of free amino acids compared to using an inert gas such as nitrogen, likely due to the associated pH reduction caused by CO_2_ dissolution in water. Regarding working pressure, its effect on the hydrolysis performance was observed to be non-significant, compared with temperature and time, as long as water remains in the liquid state. Mild reaction pressures, in the range of 40 to 60 bar, were also identified as optimal for the hydrolysis of biomass proteins to produce free amino acids (Alonso-Riaño et al. 2021).

After 300 min of treatment, the vessel was cooled, and depressurization was performed once the temperature dropped below 90 °C. Samples were then frozen at – 18 ºC until analysis and further use.

### Enzymatic hydrolysis

The commercial protease Alcalase^®^ (Novozymes [Novonesis Group], Bagsvaerd, Denmark), kindly donated by Novo Industry, was selected in this work. For the enzymatic hydrolysis, 200 mL of the WSP extract were incubated at 60 ºC and pH 8 obtained by adding NaOH. To initiate the hydrolysis, 225 U of Alcalse were added to the solution, as determined according to Pokhum et al. (Pokhum et al. 2015) with some modifications (Barea et al. 2023). After 240 min of incubation, the final hydrolysate was heated in boiling water for 5–10 min to inactivate the protease. All hydrolysates were centrifuged at 5000 rpm for 15 min to remove any dust particle from the solutions. The centrifuged solution was frozen at – 18 ºC until analysis and further use.

### Membrane filtration process

The membrane filtration studies were conducted using a dead-end stirred bench-scale stainless steel HP4750 batch cell supplied by Sterlitech Corportaion (Kent, WA, USA) with a 0.3 L capacity. The module is provided with a magnetic stirrer, a manometer, an analytical balance, and a nitrogen cylinder. The shear field generated by the stirrer helps to control membrane fouling in the dead-end module by sweeping away any dissolved matter or solute that could accumulate on the membrane surface (Roslan et al. 2018). Additionaly, it ensures uniform hydrodynamic conditions across the entire membrane surface. In any case, for each experiment, a new membrane was used to prevent any loss of permeability due to fouling. Disk samples of flat-sheet membranes were used with an effective permeate area of 14.6 cm^2^.

Four flat sheet NF membranes were tested in this work. Three flat sheet polyamide thin-film composite (PA-TFC) NF membranes: NFG, NFW, and NFX (manufactured by Synder Filtration, CA, USA) were supplied by Sterlitech. They had different molecular weight cut off (MWCO) of  ~ 600–800,  ~ 300–500 and  ~ 150–300 Da, respectively. Another NF membrane, NP010 (Microdyn-Nadir, GmbH, Wiesbaden, Germany), with a MWCO of 1–1.2 kDa, was also tested. NP010 was composed of polyethersulfone (PES) as active layer with a polyethylene/polypropylene (PE/PP) support layer. Additionally, a UF membrane, Synder-ST, with a MWCO of 10 kDa, also made of PES as the active layer, was included in the study.

At the start of each filtration experiment, fresh membranes were tested by recirculating water until no further reduction in water flux was observed. This conditioning of the membranes ensured their wettability, stabilized the permeate flux, and removed any preservatives and wetting agents from their surface, thereby enhancing the overall membrane performance.

The filtration process was carried out at room temperature with a stirring speed of 300 rpm to simulate cross-flow filtration conditions. The transmembrane pressure was 20 bar. The pressure on the permeate side was the atmospheric pressure for all the experiments conducted.

A feed solution volume of 200 mL was used for each filtration experiment. The permeate was collected continuously and weighed at regular intervals along the process. Once a certain mass concentration factor was reached, the experiment was interrumpted. Samples of the feed, permeate and retentates fractions were collected for futher analysis and stored at – 18 ºC.

**Fig. 1:**
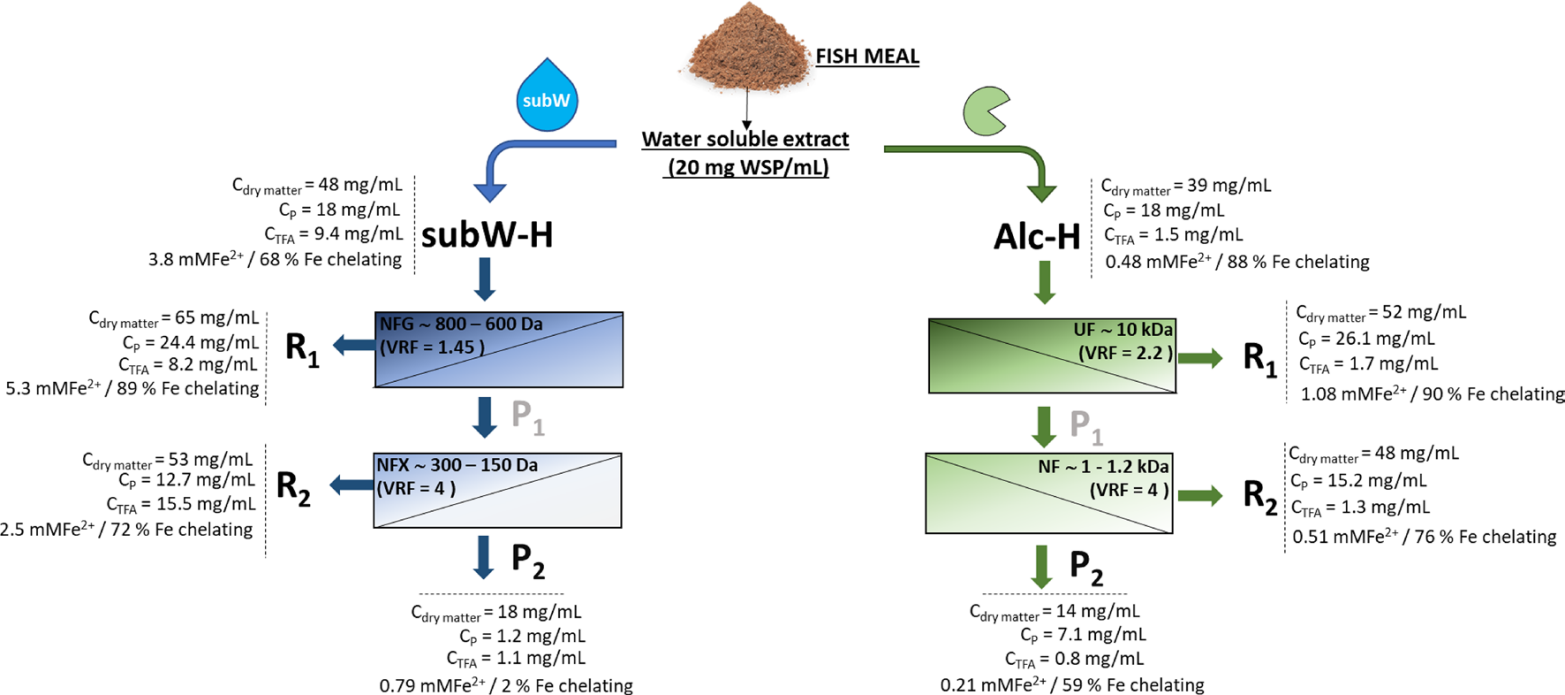
Membrane cascade approach to fractionate the protein and free amino acid fraction of subW-H and Alc-H. C_dry matter_ = dry matter concentration, C_p_ = protein concentration, C_TFA_ = total free amino acid concentration, mMFe^2+^ = reducing capacity, percentage of iron chelating capacity

Preliminary experiments were conducted using a synthetic amino acid aqueous mixture of alanine + glycine + proline, with a composition similar to that determined in the subW-H. These experiments aimed to stablish the rejection characteristics of each membrane. Based on these results and the characterization of the molecular weight distribution of the hydrolysates, a two-step sequential NF process was proposed to fractionate the subW-H, while a two-step UF/NF process was considered for the enzymatic hydrolysate (see Fig. 1). In this operation mode, at the end of the first step, the permeate recovered was used as feed solution in the second filtration step.

#### Membrane performance parameters

The permeate flux was determined by weighing the permeate collected at a specific time:

1 $$J=\frac{1}{{A}_{membrane}}\cdot \frac{{m}_{permeate}}{t}$$

where J is the permeate flux, kg·m^−2^ h^−1^, A_membrane_ is the effective membrane area, m^2^, m_permeate_ is the permeate mass, kg, and t is the filtration time, h.

The Volume Reduction Factor, VRF, is an important parameter in concentration operating mode and it is defined as:

2 $$VRF=\frac{{V}_{F}}{{V}_{R}}$$

where V_F_ and V_R_ are the initial feed and retentate volume (V_R_ = V_F_–V_P_), respectively, and V_P_ the permeate volume.

The retention percentage factor, R_i_, reflects the performance of a membrane to retain certain solutes in a hydrolysate and it was evaluated according to (Chorhirankul et al. 2024):

3 $${R}_{i}\left(\text{\%}\right)=\left(1-\frac{{C}_{P,i}}{{C}_{R,i}}\right)\cdot 100$$

The concentration factor, CF, was evaluated as:

4 $$Concentration\, factor=CF=\frac{{C}_{R}}{{C}_{F}}$$

where C_P,i_ C_R,i_ and C_F,i_ are the concentration of the solute, i, in the permeate, retentate and feed solutions, respectively.

The purity index of the peptide fraction or the free amino acids in the streams generated during fractionation, PI, was measured as the percentage of the corresponding fraction over the total dry matter content:

5 $$PI=\frac{{C}_{protein\, or\,free\,amino\, acids, stream}}{{C}_{dry matter, stream}}\cdot 100$$

The process yield, Y_i_, was defined as the percentage of a certain component entering the system in the feed stream that was recovered in the product stream:

6 $${Y}_{i }(\text{\%})=\frac{mass\, of\, i \,in \,the\, product}{mass \,of\, i\, in\, the\, feed}\cdot 100$$

The error of the mass balance, ε (i), was quantified by the relative error defined as the ratio of the mass residue of compound i when performing a mass balance around the unit operation to the initial amount of i expressed in percentage:

7 $$\varepsilon \left(i\right)=\frac{{mass}_{i, feed}-\left({mass}_{i,R1}-{mass}_{i,R2}-{mass}_{i,P2}\right)}{{mass}_{i, feed}}\cdot 100$$

### Analytical methods

#### Dry matter content

1 mL of each sample was placed in Petri dishes. The dry matter content was obtained by drying the samples in a oven at 105 ± 1 °C until constant weight was achieved. The results were expressed as mg of dry matter content/mL.

#### Total organic carbon and total nitrogen

A Total Organic Carbon Analyzer Shimadzu (TOC-V CSN) was used to quantify the concentration of total carbon (TC) and inorganic carbon (IC). Potassium hydrogen phthalate and sodium hydrogen carbonate were used as standards. The TOC concentration was then calculated by subtracting the IC concentration from the obtained TC concentration. Total Nitrogen (TN) content was determined by using KNO_3_ as standard. TOC and TN determination provides a valuable information related with the total dissolved organic carbon representing the total dissolved organic molecules to help to understand the fractionation process and chemical composition of the streams generated by evaluating the molar ratio N:C.

#### Lowry method for protein determination

The Lowry method was carried out to determine the total protein content of the different streams generated in the fractionation process (Lowry et al. 1951). Briefly, the samples were conveniently diluted to 1 mL with deionized water. 5 mL of Lowry reagent were then added (Lowry reagent: 20 g·L^−1^ sodium potassium tartrate, 20 g·L^−1^ copper sulfate pentahydrate and 20 g·L^−1^ sodium carbonate in 0.1 M NaOH; in proportion 1:1:100). Samples were incubated for 15 min in the dark. Afterwards, 0.5 mL of Folin-Ciocalteu’s phenol reagent, diluted 1:3 in distilled water, were added, and the mixture was let to stand for 30 min in the dark. Finally, absorbance was measured at 750 nm using a Jasco spectrophotometer and protein content was obtained based on a calibration curve using bovine serum albumin as standard.

#### Free amino acids

SubW-CO_2_ and enzymatic hydrolysis would yield different products after treatment. These would include partially hydrolysed proteins as peptides of different molecular weight, but also, free amino acids. The variety of these hydrolysis products will reflect the hydrolysis degree achieved by both methods. Free amino acids were determined in both subW-H and Alc-H hydrolysates as well as in the different UF and NF streams.The amino acid profile was analyzed by gas chromatography after derivatization. The analysis was performed based on the original protocol by Hušek (Hušek 1991) with some modifications. In a tube, 200 μL of the aqueous sample were mixed with 267 μL of a mixture of ethanol:piridine (4:1), 33.3 μL of propylchloroformate (PCF) as the derivatization agent and 200 μL of Norvaline 0.2 mM (as internal standard). The tube containing the sample was gently shaken twice for about 10 s each time, letting it rest for 1 min between shakes and carefully releasing the gas formed. Afterwards, 200 μL of chloroform containing 1% of PCF were added to the mixture followed by 30 s of vortex stirring to ensure thorough mixing. The derivates were transferred to the organic phase, and an aliquot of this phase was injected in GC-FID instrument (Hewlett Packard, HP 5890 Series II). Aliquots of 4 μL of the derivatized amino acids were injected at 1:15 split ratio at 250 ºC into a ZB-AAA column (Phenomenex Inc.), 10 m × 0.25 mm I.D. The initial temperature of 110 ºC was maintained for 1 min; then, the oven temperature was programmed from 110 to 320 ºC at 32 ºC/min. Helium was used as a carrier gas at 60 kPa and nitrogen was used as a make-up gas. The detector temperature was set at 320 ºC. Amino acids were identified using pure standards for the different amino acids and with norvaline as internal standard.

The amino acid profile of the WSP was determined previous hydrolysis of the soluble protein before the derivatization. Acid hydrolysis was carried out using 6 N HCl at 100 °C for 24 h. Tryptophan, cysteine and methionine are partially or totally destroyed on this acid hydrolysis, so an alkaline hydrolysis was also carried out to determine these amino acids using a 4.2 M NaOH for 24 h at 110 ºC (Barea et al. 2023).

#### Determination of reducing and iron chelating capacity

Reducing capacity was assessed by the Ferric Reducing Antioxidant Power (FRAP) assay according to Benzie and Strain (Benzie and Strain 1996). A solution of FeSO_4_⋅7 H_2_O (0.1 M) was used as standard. Results were expressed in μmoles Fe^2+^/L.

The metal chelating activity was determined according to the method described by Ketnawa et al. (Ketnawa et al. 2016). 800 µL of the liquid samples were mixed with 10 µL of iron dichloride solution (2 mM) and 20 µL of Ferrozine (5 mM). After keeping the mixture for 10 min at room temperature, the absorbance was measured at 562 nm. The blank (sample with 30 µL of water instead of reagents) was substracted from the measured absorbance and the result was compared with the control measurement (water instead of sample). Results were expressed in % of iron chelating capacity.

#### Size exclusion chromatography

The molecular size distribution of the different samples was measured by Gel-Permeation Size Exclusion Chromatography coupled to a refraction index detector (GPC-SEC-RID, 1260 HPLC system, Agilent Technologies, CA, USA). The column system consisted of a Proteema precolumn (4.6 × 30 mm) and a micro column in series (4.6 × 250 mm) with a porosity of 100 Å and a particle size of 3 μm (PSS Polymer Standards Service GmbH), which allowed separation in the range from 100 to 150000 Da. Characterization of samples was performed in isocratic mode with 0.01 M NH_4_Ac, at a flow rate of 0.3 mL/min at 35 °C. Standards used for the calibration consist of a pullulan standard set (342 – 343000 Da). Data were analyzed with Agilent OpenLab Data Analysis 2.5 software. After filtration through 0.45 µm syringe filters, a volume of 10 μL of samples and standards were injected. The total area of the chromatogram was integrated and separated into fractions of five molecular weight (MW) ranges (> 10000, 10000–6000, 6000–4000, 4000–2000, 2000–1000, 1000–300, and < 300 Da, respectively), expressed as the percentage of the total area.

## Results and discussion

### Production and characterization of subcritical water and enzymatic hydrolysates from the water soluble protein fraction

The production of the WSP fraction from tuna fish meal, as detailed in Sect. “Water soluble protein extraction”, resulted in an aqueous solution with a concentration of 20.5 ± 0.8 mg of WSP/mL, as determined by the Lowry assay. subW-CO_2_ hydrolysis and enzymatic hydrolysis were carried out to generate small peptides and free amino acids in the medium. This hydrolysis process was a crucial step that determined further design strategies for the stages of separation, fractionation, and purification of small peptides and the free amino acids generated.

The amino acid profile of the subW-H and the Alc-H is summarized in Table 1. Additionally, the amino acid profile of the WSP is also presented in Table 1, being glycine (199 ± 9 mg/g_wsp_), proline (111 ± 5 mg/g_wsp_), glutamic acid (including glutamic acid and glutamine, 103 ± 14 mg/g_wsp_) and alanine (83 ± 7 mg/g_wsp_) identified as the most abundant amino acids in the WSP fraction of fish meal.

**Table 1:** Amino acid profile of the WSP from fish meal. Characterization of the subW-H at 180 ºC and Alc-H at 60 ºC: Free amino acid profile, total protein content, dry matter content and reducing and chelating capacity

Parameter	WSP	subW-H	Alc-H
Amino acids, aa	aa profile, mg aa/g_WSP_^1^	Free amino acids released in the hydrolysate, mg/mL	
ALA	83 ± 7	1.55 ± 0.05	0.135 ± 0.003
GLY	199 ± 9	3.05 ± 0.07	0.067 ± 0.006
VAL	29 ± 5	0.22 ± 0.02	0.054 ± 0.005
LEU	36 ± 2	0.19 ± 0.3	0.07 ± 0.01
ILE	18 ± 2	0.09 ± 0.01	0.041 ± 0.004
TRE	31 ± 2	0.074 ± 0.005	0.024 ± 0.004
SER	36 ± 5	0.14 ± 0.02	0.033 ± 0.006
PRO	111 ± 5	1.04 ± 0.07	0.047 ± 0.005
ASP	70 ± 7	0.94 ± 0.1	0.12 ± 0.02
MET	12 ± 2	0.15 ± 0.01	0.044 ± 0.009
HYP	58 ± 9	0.39 ± 0.08	0.021 ± 0.009
GLU	103 ± 14	0.30 ± 0.03	0.06 ± 0.02
PHE	21 ± 2	0.32 ± 0.04	0.083 ± 0.07
LYS	37 ± 6	0.24 ± 0.02	0.13 ± 0.03
HIS	13 ± 2	0.46 ± 0.03	0.33 ± 0.01
HLY	51 ± 18	0.062 ± 0.005	0.086 ± 0.003
TYR	15 ± 3	0.082 ± 0.003	0.06 ± 0.01
TRP	54 ± 12	0.034 ± 0.005	0.042 ± 0.003
CYS	25 ± 9	0.057 ± 0.004	0.05 ± 0.01
Total	1002 ± 121	9.4 ± 0.9	1.5 ± 0.2
Dry matter, mg/mL	40 ± 2	48 ± 3	39 ± 1
Protein, mg/mL	20 ± 1	18 ± 1	18.6 ± 0.7
mM Fe^2+^	0.57 ± 0.01	3.8 ± 0.2	0.48 ± 0.01
Chelating capacity, %	5.6 ± 1.3	68 ± 2	88 ± 1

ALA: alanine, GLY: glycine, VAL: valine, LEU: leucine, ILE:isoleucine, TRE: threonine, SER:serine, PRO: proline, Asp: aspartic acid and asparragine, MET: methionine, HYP: hydroxyproline, GLU: glutamic acid and glutamine, PHE: phenylalanine, LYS: lysine, HIS: histidine, HLY: hydroxylysine, TYR: tirosine, TRP: triptophan, CYS: cysteine

^1^20.5 ± 0.8 mg protein/mL

subW-CO_2_ and enzymatic treatments yielded hydrolysates with different properties and chemical composition (Barea et al. 2023). These authors showed the generation of peptides of smaller size by subW-CO_2_ compared to enzymatic hydrolysis as supported by size exclusion chromatography. subW-H yielded a higher amount of free amino acids (9.4 ± 0.9 mg/mL) compared to Alc-H (1.5 ± 0.2 mg/mL, see Table 1). Table 2 summarized the molecular weight (MW) distribution of both hdryolysates together with the MW distribution of the WSP. It can be observed that the Alc-H contains peptides with a higher degree of polymerization, accounting for 38.5% (w/w) peptides with MW higher than 4000 Da, while this percentage was 14.1% (w/w) for subW-H. It must be also highlighted the high percentage of molecules with a MW lower than 300 Da in the subW-H according to the higher amount of free amino acids (Table 1). In any case, the percentage of peptides with MW higher than 4000 Da was lower in both hydrolysates than the distribution determined for the initial WSP extract with more than 60% (w/w), 37.7% (w/w) corresponding to the fraction of MW > 10.000 Da. The different chemical composition determined the selection of the membrane separation cascade processes to concentrate the valuable fractions of the protein hydrolysates (see Fig. 1).

**Table 2:** Molecular weight distribution (Da) of the WSP extract, subW-H and Alc-H and their UF/NF fractions, expressed in w/w %

	> 10000	10000–6000	6000–4000	4000–2000	2000–1000	1000–300	< 300
WSP extract	37.7 ± 0.3	12.5 ± 0.2	10.0 ± 0.1	26.4 ± 0.3	8.4 ± 0.2	3.7 ± 0.1	1.30 ± 0.05
subW-H	0.20 ± 0.05	10.5 ± 0.4	3.4 ± 0.1	18.7 ± 0.3	13.1 ± 0.3	15.9 ± 0.2	38.1 ± 0.4
NF, R1	2.1 ± 0.1	21.7 ± 0.3	4.6 ± 0.1	20.1 ± 0.2	9.5 ± 0.2	13.3 ± 0.3	28.6 ± 0.4
NF, R2	0.01 ± 0.00	7.7 ± 0.2	2.9 ± 0.1	17.4 ± 0.2	18.4 ± 0.3	15.7 ± 0.3	37.8 ± 0.5
NF, P2	0.00 ± 0.00	0.00 ± 0.0	0.00 ± 0.00	6.6 ± 0.1	23.7 ± 0.4	44.8 ± 0.5	24.8 ± 0.3
Alc-H	11.6 ± 0.2	16.8 ± 0.2	10.1 ± 0.2	30.2 ± 0.4	19.4 ± 0.4	5.1 ± 0.2	6.7 ± 0.1
UF, R1	14.2 ± 0.3	21.0 ± 0.4	10.0 ± 0.1	24.2 ± 0.4	19.8 ± 0.3	5.0 ± 0.1	5.8 ± 0.2
NF, R2	5.1 ± 0.2	14.0 ± 0.3	9.4 ± 0.2	34.4 ± 0.5	23.7 ± 0.4	5.7 ± 0.1	7.7 ± 0.2
NF, P2	2.3 ± 0.1	6.8 ± 0.1	14.8 ± 0.3	49.4 ± 0.4	10.8 ± 0.2	6.2 ± 0.2	9.6 ± 0.3

A higher reducing capacity was observed for the subW-H compared to the Alc-H, 3.8 ± 0.2 and 0.48 ± 0.01 mMFe^2+^, respectively. However, the Alc-H exhibited higher iron chelating capacity with a value of 88 ± 1% compared to 68 ± 2% for subW-H. It is important to note that the FRAP reducing capacity test is based on single electron transfer reactions; while transient metal ion chelation acts indirectly as an antioxidant mechanism by inhibiting radical chain reactions. The higher temperatures of subW-CO_2_ treatment compared to enzymatic hydrolysis could led to conformational changes of peptides hydrolysates leading to lower iron chelating capacity, although presenting peptides with low molecular weight distribution (Barea et al. 2024). Furthermore, the lower iron chelating capacity determined for subW-H could also be related with the higher amount of free amino acid released into the medium. Acording to Guidea et al. (Guidea et al. 2020), metal chelating capacity of the free proteinogenic amino acids shows a moderate Fe^2+^ chelating activity with values of 4.90 ± 0.03, 65 ± 1 and 34 ± 3% for the major free amino acids released by subW-CO_2_ treatment: alanine, glycine and proline (see Table 1), respectively. It is important to note that Guidea et al. (Guidea et al. 2020) achieved these results by using standard amino acid solutions in known concentrations, in contrast to the complex matrices employed in this study.

### Membrane concentration process in stirred dead-end filtration

#### Pure amino acid mixture filtration

**Fig. 2:**
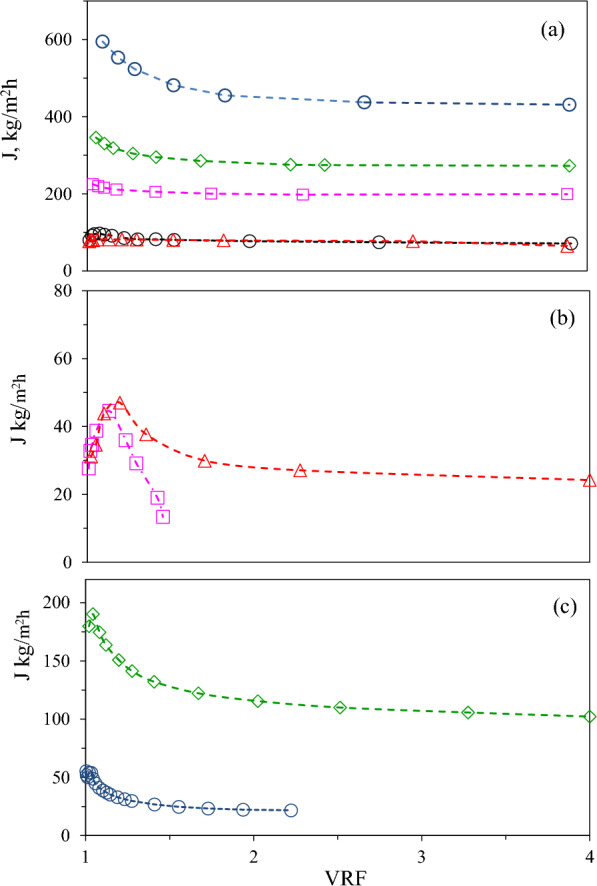
Total permeate flux (J) for (**a**) pure amino acid mixtures ( UF-Synder,  NP010  NFG  NFW,  NFX). **b** subW-H ( 1st step NFG,  2nd step NFX). **c** Alc-H ( 1st step UF-Synder,  2nd step NP010). Lines are to guide the eye

First, the effectiveness of different NF membranes in retaining the major free amino acids released during subW-CO_2_ treatment was studied. The feed amino acid concentrations were as follows: 1.5 ± 0.1 mg alanine/mL, 3.0 ± 0.1 mg glycine/mL ± 0.1 and 1.0 ± 0.1 mg proline/mL. After conditioning the membranes with water, permeation of the multicomponent pure amino acid mixture was performed with the four NF membranes used in this work (NFG, NFW, NFX of polyamide-TFC and NP010 of PES). Filtration experiments were ended at a VRF = 4. Additionaly, the amino acid retention for the UF-ST membrane was also tested for the pure amino acid mixture to cover all the membranes considered in this study. Figure 2a shows the permeate flux determined for the five membranes. The UF Synder ST membrane exhibited the highest permeate flux with an abrupt decay observed at the beginning of the process, 23% of decrease, followed by a plateau around 2 VRF values. The total permeate flux for all the NF membranes was lower than for UF and there was observed a correlation between MWCO and membrane permeate flux for NF regardless the membrane material (PES or PA-TFC). However, it must be highlighted that only slightly higher permeate flux were determined for NFX (300–500 Da) compared to NFW (150–300 Da). There was also observed an initial decay in J for the NF membranes with the highest MWCO (NFG and NP010), similar to the pattern observed for the UF-ST; with 17 and 9% of decrease for NP010 and NFG, respectively. On the contrary, the permeate flux exhibited a smooth maximum for NFW and NFX, followed by a slight decrease. Tamires et al. (Tamires Vitor Pereira et al. 2020) also observed a similar phenomenon for different NF membranes, where the permeate flux increased at the beginning of the filtration process, stabilizing after a certain time. This behaviour was attributed to the swelling of the membrane, which provided greater flux initially and resulted in less rejection by the membrane.

**Fig. 3:**
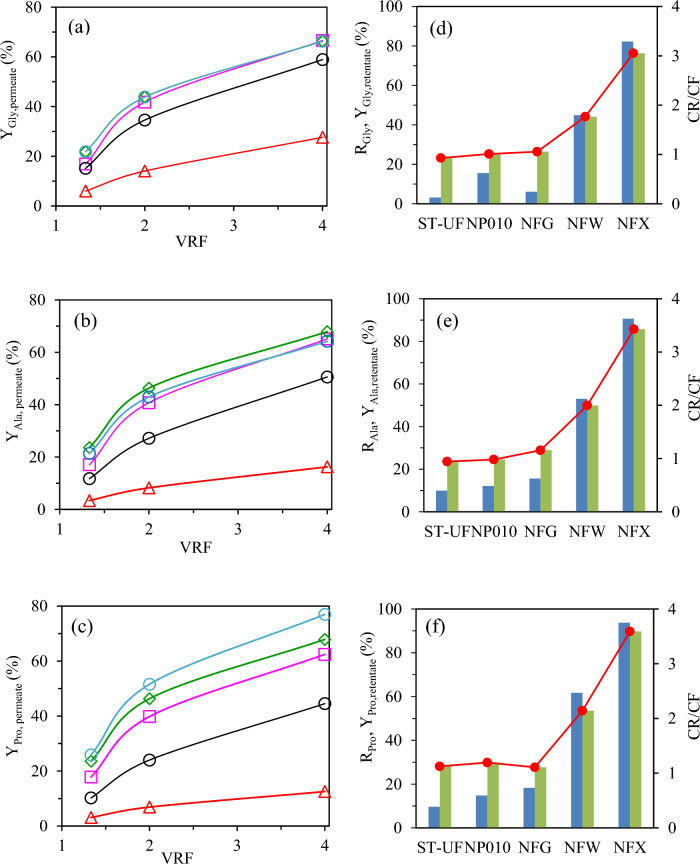
**a**, **b**, **c** Percentage recovery of the amino acid in the permeate stream as a function of the concentracion factor (**a**) glycine (**b**) alanine (**c**) proline for different membranes:  UF-Synder,  NP010  NFG  NFW,  NFX). **d**, **e**, **f** Amino acid retention (), percentage recovery in the retentate stream () and CR/CF() at VRF = 4 for the different membranes

At various VRF ratios, permeate samples were collected and analyzed for amino acid composition. A steady increase in the total amount of amino acids permeating through the membrane was observed as the value of the VRF ratio increased for the three amino acids and for each of the membranes studied (Figs. 3a–c), being this increase less pronounced for the smallest pore size membrane, NFX.

A correlation was also observed between the MWCO for the series of NFG/NFW/NFX membranes and amino acid permeation, with higher permeation for all the three amino acids as the MWCO of the membrane increased. However, for the NP010 and UF membrane of PES polymer, there was no clear correlation between MWCO and permeation of the three amino acids. In fact, similar percentages of the amino acids in the permeate stream were obtained for the NP010 membrane (1–1.2 kDa), the Synder-UF (10 kDa), and of the order of NFG membrane, specially for the smallest amino acids, alanine and glycine, probably due to the small molecular weight of these amino acids compared to the MWCO of these membranes. This was also reflected in the values of the retention percentage factor (R_aa_ %) of the amino acids (Eq. 3) and the yield of the amino acid in the retentate stream (Y_aa,retentate_ %) (Fig. 3d–f). For example, at VRF = 4.0, for the 600–800 Da NFG membrane, R_glycine_, the smallest amino acid, was 6%, increasing to 45 and 82% for the 300–500 Da NFW and 150–300 Da NFX, membranes respectively, corresponding to values of Y_glycine,retentate_ of 26, 44 and 76%. The values for NP010 and UF membranes were of similar order as for the NFG membrane. These results support the idea that the amino acid separation occurs not only by size exclusion, but also by the interaction of the membrane polymer with the components of the feed, which might play an important role, as suggested by Chorhirankul (2024) in their study. For instance, values as high as 23, 24 and 28% for Y_glycine,retentate_, Y_alanine,retentate_ and Y_proline,retentate_ were obtained for 10 kDa UF-Synder membrane (MWCO >  >  >  > M_w,amino acids._)_._

The concentration factor, CF (Eq. 4), was also plotted for alanine, glycine and proline at the end of the process (VRF = 4) for all the membranes. According to the previously presented values, CF values significantly higher than the unity were only obtained for NFW and NFX membranes. Therefore, NFW or NFX membranes could be appropriate for amino acid concentration, and considering the higher retention values for all the amino acids, NFX membrane could be a good option as NF step to concentrate the high amount of free amino acid obtained in the subW-H, previous removal of larger peptides.

Additionaly, a correlation was also observed between the molecular weight of the three amino acids and their corresponding retentions for each individual membrane (Fig. 3). For instance, for the NFX membrane at VRF = 4, R_Glycine_ = 82%, Y_Glycine,retentate_ = 76% and CF = 3.1, were obtained for glycine (M_W_ = 75.07) while these parameters were evaluated as R_Alanine_ = 91%, Y_Alanine,retentate_ = 86% and CF = 3.4 for alanine (M_W_ = 89.09); and R_Proline_ = 94%, Y_Proline,retentate_ = 90% and CF = 3.6 for proline (M_W_ = 115.13). A similar pattern was observed for the other membranes with higher MWCOs (Fig. 3), but with lower values of the retention, amino acid recovery in the retentate and concentration factor, according to their increased permeation through the membrane.

Mass balances were performed for each amino acid, considering the initial mass in the feed and the collected mass in the retentate and permeate streams for all the tested membranes at the end of the filtration experiment (VRF = 4). The relative error in the mass balance was consistently lower than 10%, except for experiment with UF membrane that reached deviations of 12 and 10% for alanine and glycine, respectively.

#### ***Two-step nanofiltration process to fractionate subW-CO***_***2***_*** hydrolysates***

##### Permeate flux

Based on the results obtained from the molecular weight distribution of the subW-H and the separation of a pure amino acid mixture, a two-step NF process was proposed to fractionate the components of this hydrolysate (Fig. 1). Initially, the NFG membrane (∼600–800 Da) was selected to retain the fraction of molecules in the subW-H with MW higher than 800 Da, which approximately corresponds to 46 % of the total molecules in the hydrolysate (considering the molecules of MW higher than 1000 Da, as shown in Table 1). Permeate from this first step is expected to be enriched in the free amino acids present in the subW-H. Therefore, the permeate obtained from this initial fractionation step was used as feed in a second filtration step using the NFX membrane. The NFX was chosen over the NFW membrane based on results obtained for filtration of a pure amino acid synthetic mixture, where both membranes (NFX ~150–300 Da and NFW ~300–500 Da) showed similar permeate flux. However, the NFX membrane provided higher retention and concentration factors for individual free amino acids. The polyamide membrane series was considered in the subW-H fractionation to maintain consistency in the polymer type throughout the NF sequence for this hydrolysate.

The permeate flux has been plotted in Fig. 2b showing much lower values compared to the synthetic mixture of amino acids (Fig. 2a), specially for NFG membranes. The initial permeate flux (J_o_) for NFG membrane was 225 kg/m^2^h in the amino acid mixture decreasing to 28 kg/m^2^h for subW-H. This result suggests that fouling on the membrane surface has a significant effect on permeation performance; peptides and free amino acids can be adsorbed on the poliamide membrane reducing the permeate flux (Tamires Vitor Pereira et al. 2020).

In the 1st stage, larger solutes with diameters larger than the membrane pores could be deposited on the membrane surface, causing the formation of a cake layer. Additionally, solutes of similar size to the pore size of the membrane could also partially seal the membrane pores. Due to low and continuously decreasing permeate flux, the experiment was stopped when the VRF reached 1.45, with a permeate flux of 13.4 kg/m^2^h that corresponds to 0.33 g/min. This was before reaching the VRF of 4.0, which was studied for the pure amino acid mixture and would have resulted in an extended operation time. In the 2nd step of the sequential process, the decrease in the permeation flux for the NFX membrane was less pronounced, with J_o_ values of 77 kg/m^2^h and 31 kg/m^2^h for the amino acid mixture and the hydrolysate, respectively, at a VRF of 4.0 in this second step. The lower J decrease in permeation flux in the 2nd step was observed in other sequential processes, as reported by Saidi et al. (2013) in their study, due to the reduction of adsorption on the membrane surface, which is related to the lower amount of dry matter in the feed of the 2nd stage (P1). The initial content of dry matter in the subW-H (feed of the 1st stage) was 48 mg dry matter/mL (Fig. 1), while this concentration was reduced to 26 mg dry matter/mL in the P1 (feed of the 2nd stage). This low dry matter concentration in the 2nd stage allowed reaching a VRF of 4 in the second stage. Dry matter content was also determined in the R1, R2 and P2 streams, being 65, 53 and 18 mg/mL, respectively. The dry matter concentration allowed us to determine its retention percentages in the two consecutive stages, considering the corresponding feed entering the system as 61 and 67% in the 1st and 2nd stages, respectively. For both membranes, the permeate flux initially increased from 28 to 45 kg/m^2^h for NFG membrane and from 31 to 47.0 kg/m^2^h for NFX membrane, and then decreased continuously until the end of the process. Swelling of the membrane could initially lead to higher flux, allowing more soluble solids to adhere to the membrane surface in a given time period. This could result in a faster growth of the cake layer that led to faster decrease in permeate flux, specially for the NFG membrane.

**Fig. 4:**
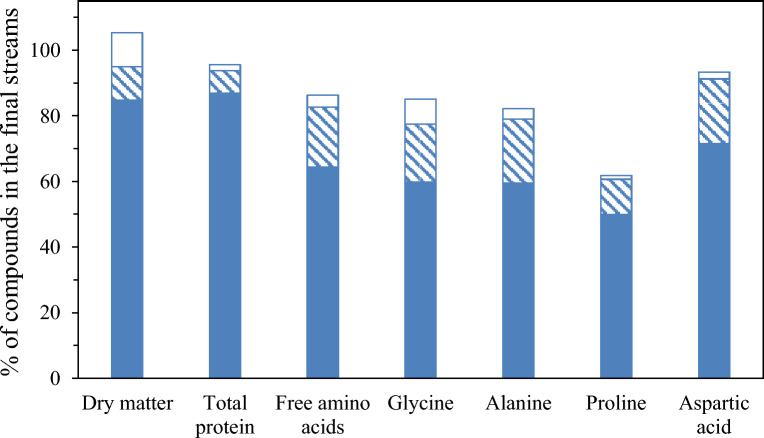
Mass balance of dry matter, protein content, free amino acids and the major free amino acids determined in subW-H (retentate of the NFG step ( R1); retentate of the NFX step ( R2) and permeate of the NFX step ( P2)

Mass balance of the total dry matter has been plotted in Fig. 4 considering the final streams R1, R2 and P2 of the consecutive process. At the end of the sequential step, most of the dry matter was retained in the first retentate (yield of 84.7%), while only 10.3% of the initial dry matter content of the feed was in R2 and P2. This is consistent with the low permeate flux in the first stage. The error of the mass balance, evaluated with Eq. 7, was determined as 5.3% (Fig. 4).

##### Peptides and free amino acid separation

**Fig. 5:**
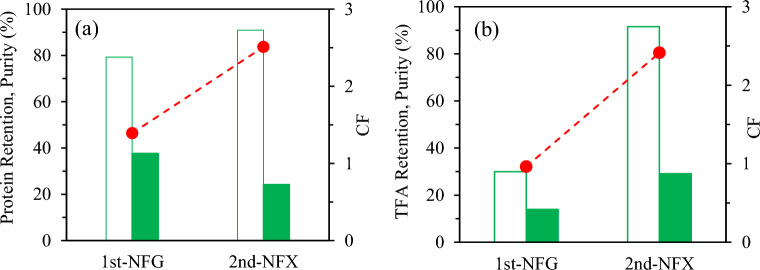
Retention percentages, purity and concentration factor of subW-H (**a**) Peptide fraction (**b**) Total Free amino acids (TFA). ( retention percentage, purity index in the retentates,  concentration factor)

**Peptides.** During the fractionation step, the protein concentration in the R1 (VRF = 1.45) increased up to 24.4 mg/mL (CF = 1.4, from 18 mg/mL). The highest protein concentration factor was reached in the second NF step increasing from 5.1 mg/mL (P1 = F2) to 12.7 mg/mL (CF = 2.5). These results indicated that most of the protein fraction was rejected by the membranes and concentrated in the retentates in the two-step consecutive process. Figure 5a collects the retention percentages evaluated with the reported concentration data of the protein fraction, yielding 79% and 91% in the 1st and 2nd stages and purity indexes of 38% and 24%, in R1 and R2, respectively.

The mass balance of total protein content was determined and also plotted in Fig. 4, showing that 87% of the initial protein content was retained in the R1 and less than 2% of the initial protein content was determined in the final permeate P2. The error of the mass balance, evaluated with Eq. 7, was less than 4.5%, indicating that the material balance closely matches for the protein fraction and that it was not adsorbed on a great extent on the surface of the poliamide membranes.

**Total free amino acids.** The retention percentage, purity and concentration factor of total free amino acids in the subW-H have been plotted in Fig. 5b. Total free amino acid content was evaluated as the sum of the individual free amino acids as determined by gas chromatography (Sect. “Production and characterization of subcritical water and enzymatic hydrolysates from the water soluble protein fraction.”). The retention percentage in the 1st stage was 30%. This value is slightly higher than the value previously reported for the individual amino acid in a synthetic pure amino acid mixture, which could be attributed to the a higher fouling degree observed during the subW-H filtration in this first step. On the contrary, the rentention degree of total free amino acids in the 2nd stage (NFX membrane) was 92%, within the range of values reported for individual amino acids in the synthetic mixture. The purity index for total free amino acids was 14 and 29% in the retentates of the consecutive steps, R1 and R2, respectively, achieving a concentration factor of 2.4 in the second step.

**Fig. 6:**
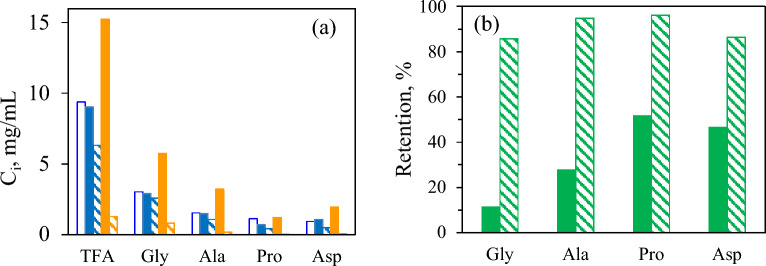
(**a**) Concentration of TFA and some individual free amino amino acids in the different streams  subW-H,  R1;  P1;  R2;  P2. (**b**) Retention percentage of most abundant free amino acids in subW-H (R1, R2) Gly = glycine, Ala = alanine, Pro = proline, Asp = aspartic acid

Table 1 reports the individual free amino acids determined in the subW-H, with alanine, glycine and proline being the major free amino acids released. To evaluate the behaviour of these individual amino acids, Fig. 6a plots the concentration of these individual free amino acids in the different streams, along with the concentration of the total free amino acids. This Figure also includes aspartic acid since its percentage in the initial feed was 10.8% of the total free amino acids (0.94 mg/mL, Table 1), slightly lower than that of proline. The individual retention degree in each stage followed a similar pattern to the retention determined for the total free amino acids (see Fig. 5b), with retention values higher than 85% in the second stage of the consecutive process. For the smallest amino acids (glycine and alanine) the lowest value for the retention percentage was reached in the first stage, with values of 11.3 and 27.7%, respectively.The highest concentration of total free amino acids was determined in the R2 (15.3 mg/mL), with glycine (5.8 mg/mL) and alanine (3.2 mg/mL) being the most abundant amino acids in R2, accounting both amino acids for 59% of the total free amino acids in this stream.

The mass balance of total free amino acids and the selected individual free amino acids was evaluated and also included in Fig. 4. 64% of the initial total free amino acid content was retained in the R1, 18% in R2 and less than 4% was determined in the final permeate P2. The error of the mass balance, evaluated with Eq. 7, yielded a higher error in the mass balance compared to the total protein fraction and the dry matter, with an error of 13.7%. The errors in the mass balance for glycine, alanine and aspartic acid were of the same order (Fig. 4); howewer, the mass balance was not satisfactory for proline, which could be due to analytic problemas (specially in the permeate fraction where its concentration was very low) or to adsorption on the membrane materials.

**Total organic carbon and nitrogen** as determined by TOC-Analyzer, was evaluated in the different streams of this consecutive process. The values obtained allowed us to evaluate the molar ratio N:C in the different streams. The ratio N:C was in the range of 0.31–0.32 for the feed (subW-H) and R1 and R2 fractions. These values agree with the N:C ratio reported by Rivas-Ubach (Rivas-Ubach et al. 2018) for peptides-type compounds (N:C = 0.3) indicating that most of the organic matter corresponds to protein-type compounds, which aligns with the chemical composition of the initial extract of WSP. However, the N:C ratio in the final permeate P2 was higher, N:C = 0.49. This higher value could be consistent with the higher amount of glycine (C_2_H_5_NO_2_, N:C = 0.5) in the final permeate compared to other peptides and free amino acids.

**Molecular weight distribution.** The MWD of the three diferent final streams (R1, R2 and P2) generated in the two consecutive steps are listed in Table 2. As described in Sect. “Production and characterization of subcritical water and enzymatic hydrolysates from the water soluble protein fraction.” the weight percentage of peptides of MW higher than 4000 Da accounted only for 14.1% in the subW-H, with only 0.2% of MW higher than 10 kDa, due to higher hydrolytic power of the subW reaction medium due to the addition of CO_2_. These higher MW peptides were concentrated in the R1 fraction, as this fraction accounted for 28.4% (w/w) of the distribution (21.7% in the range from 10 to 6 kDa). The significant flux reduction determined in the first step could therefore be related to the substantial membrane fouling due to the presence of these peptides of higher molecular ranges. However, R1 still contained a large peptide fraction with MW lower to the MWCO of NFG (600–800 Da, Table 2). On the contrary, the percentage of peptides with MWCO higher than 4 kDa in R2 was 10.7%, less than half of the percentage of R1. The lower concentration of these high MW molecules supports the fact that the permeate flux in the second step was relatively higher than in the first step. Peptides with MW below 4000 Da accounted for 89.3 and 100% in R2 and P2, respectively. However, the NFX retentate (R2) still contained peptides of MW below 300 Da, while peptides higher than 300 Da were also presented in the permeate (P2). Both membranes, NFG and NFX, initially swelled upon contact with the hydrolysate (see Fig. 2b) that could lead to the permeation of peptides of higher MW than the MWCO of the membrane.

Although a certain trend was observed with the MWCO of the different streams, results indicate that the selected membranes did not achieved sharp separations, since a relative wide MW distribution of peptides was found in several fractions. Similar conclusions were drawn by Picot et al. (Picot et al. 2010) in the treatment by UF and NF in a consecutive process to fractionate Prolastin, a commercial hydrolysate obtained by controlled proteolysis of skins from North Atlantic lean fish. These authors observed that a first UF step with a modified polyethersulfone membrane (MWCO of 4 kDa) still produced a retentate with a large amount of peptides below 4000 Da (as high as 93% by weight), even though high VFR was reached. Meanwhile, the NF retentate of the second setp process proposed by Picot et al. (Picot et al. 2010) still contained about 22% of peptides below 300 Da (MWCO of the membrane).

##### Reducing and iron quelating capacity

**Fig. 7:**
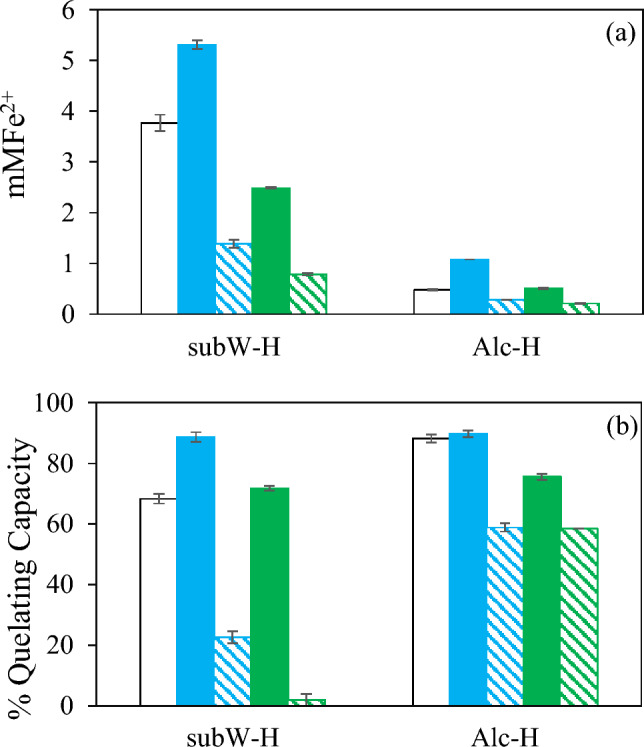
(**a**) Reducing capacity (**b**) Iron quelating capacity of subW-H and Alc-H in the different streams  initial hydrolysate,  R1;  P1;  R2;  P2

The reducing capacity, expressed as mMFe^2+^ and the iron quelating capacity of the different streams generated in the cascade process have been plotted in Fig. 7. Both parameters followed a similar trend in the different streams. R1 and R2 presented higher reducing and iron quelating capacity than the corresponding permeates, with the highest value determined in the R1, 5.30 mMFe^2+^ and 89% of iron quelating capacity. The higher reducing capacity observed in the feed (F) and R1 could be due to a synergistic effect among the different peptides generated in the hydrolysis process, reducing this effect when the membrane fractionation was performed and peptides of higher MW were retained in the R1 fraction. There was a strong positive correlation between both, reducing and iron quelating capacities (r = 0.9103, p-value = 0.0318 < 0.05), according to the Pearson linear correlation. Furthermore, both parameters were visually correlated with the color of the different fractions; the darker the fraction, the higher the reducing and iron quelating capacity.

The lower reducing capacity for P1, R2 and P2 could be also attributed to its lower dry matter content. The following values of reducing capacity were obtained when expressing the results as mMFe^2+^/mg_DM_: 0.079, 0.082, 0.054, 0.047, and 0.044 for F, R1, P1, R2 and P2, respectively. When evaluating the reducin capacity as mMFe^2+^/mg_protein_ the following values for F, R1, P1, R2 and P2 were obtained: 0.21, 0.22, 0.28, 020, and 0.66, respectively, showing the highest value for P2, free of molecules of MW higher than 4000 Da.

#### Fractionation of Alcalase^®^ hydrolysates

##### Permeate flux

Alc-H presented a low concentration of free amino acids (1.5 mg/L) with a peptide fraction of MW higher than 10 kDa of 11.6% of the total peptide fraction (16.8% close to the MWCO of the membrane, in the range of 10000–6000 Da, according to the distribution presented in Table 1). Based on this composition, the following cascade approach was proposed: (1) a 1st UF step by using the UF-Synder membrane with a MWCO of 10 kDa to remove the peptide fraction of higher MW, (2) the permeate from the UF step will be the feed of a 2nd NF step by using the NP010 membrane (Fig. 2) to concentrate molecules with MW higher than 1000–1200 Da but lower than 10,000 Da. Due to the low concentration of free amino acids in the Alc-H, no concentration step was proposed for this fraction. The NP010 membrane was chosen over the NFG membrane to use the same polymer throught the Alc-H fractionation sequence, specifically polyethersulfone polymer.

The permeate flux for both separation steps has been plotted in Fig. 2c. A great reduction in the permeate flux was obtained when using the Alc-H compared to the amino acid mixture as feed, from 594 to 52 kg/m^2^h for UF-Synder and from 346 to 180 kg/m^2^h for NP010, respectively. Surprisingly, J was lower in the 1st step for UF-Synder (10 kDa) than in the 2nd separation step using NP010 NF membrane (1–1.2 kDa). This could be attributed to the larger pore size of UF-Synder membrane making it more susceptible to pore blocking and particle deposition, resulting in a greater permeate flux decline. However, fouling is a complex phenomenon depending on the characteristics of the membrane and the feed. Polyethersulfone membranes could be classified as hydrophobic when compared with polyamide membranes. In the literature, it has been described that peptides usually adsorb more readily to hydrophobic materials compared to hydrophilic ones (such as NFG and NFX) (Chorhirankul et al. 2024) which could lead to the observed high initial flux decrease for UF-Synder due to the formation of a dense cake layer by the peptides in the feed. Due to complex fouling processes observed during the first UF step, filtration was stopped when the VRF reached 2.2, corresponding to a permeate flux of 21.7 kg/m^2^h, 0.53 g/min, to avoid prolonged operation times.

A less pronounced decrease in J was observed in the second separation step, compared to what was observed in the subW-H fractionation. The higher permeate flux obtained with NP010 could be explained by considering the dry matter content in the different streams, similar to the performance of subW-H. Dry matter in the feed, the Alc-H, was 39 mg/mL, while the dry matter concentration of the P1 stream (the feed of the 2nd stage) was 25 mg/mL. The dry matter retention coefficients were 52 and 71% in the 1st and 2nd stages, respectively.

**Fig. 8:**
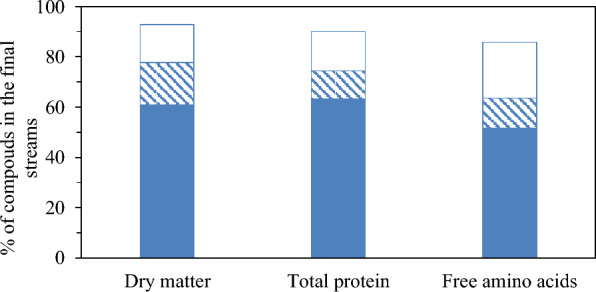
Mass balance of dry matter, protein content and free amino acids in Alc-H (retentate of the UF step ( R1); retentate of the NP010 step ( R2) and permeate of the NP010 step ( P2)

The values of dry matter concentration and VRF allowed us to evaluate the mass balances for the total dry matter considering the three final streams obtained in the process: R1, R2 and P2 (Fig. 8). The yield for dry matter in the R1 stream was 61%. This high retention of the dry matter in the first step also explains the low J values. This value was lower than the yield for subW-H due to the higher MCWO of the UF membrane compared to that of the NFG membrane employed in subW-H. Similar dry matter yields were obtained in the R2 and P2 fractions in the second fractionation steps, with values of 17 and 15%, respectively. The error of the mass balance, evaluated with Eq. 7, was determined to be 7%.

##### Peptides an amino acid separation

**Fig. 9:**
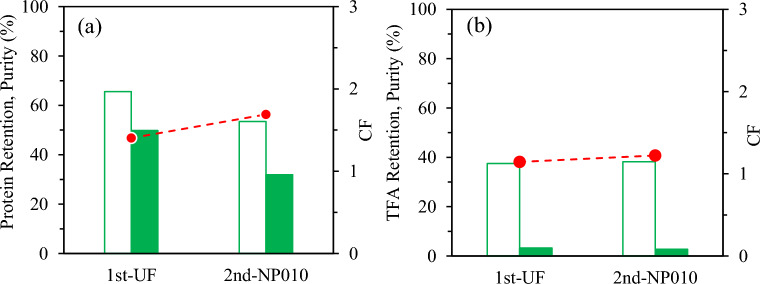
Retention percentages, purity in the retentates and concentration factor of Alc-H for (**a**) for the peptide fraction, and (**b**) Total free amino acids (TFA). ( retention percentage,  purity index,  concentration factor)

**Peptides** The protein concentration was determined in the different streams generated in the consecutive fractionation steps for the Alc-H with values of 26.1, 9.0, 15.2 and 7.1 mg/mL in the R1, P1 (P1 = F2), R2 and P2 streams, respectively (CF_1st step_ = 1.4 and CF_2nd step_ = 1.7). The corresponding retention percentages for each stage have been plotted in Fig. 9a. The protein retention percentages were lower than the values achieved for the subW-H by two consecutive NF steps. The higher MWCO of the UF-Synder membrane allowed a higher permeation of the protein fraction, although molecular weight distribution of Alc-H showed peptides with higher MW, yielding a moderate value of the retention percentage, 66% (purity index 50%). The second step yielded a retention percentage of the protein fraction of 54% (purity index of 32%) (Fig. 9a). Therefore, there was still an important fraction of the protein content with MW lower than 1–1.2 kDa that would permeate through the NP010 NF membrane. This result can also be observed in the mass balance of the total protein fraction plotted in Fig. 8. Most of the initial protein content was retained in R1, 63%, but still 16% was determined in the final permeate (P2), while 11% of the initial protein fraction was retained in the R2 stream. The error of the mass balance, evaluated with Eq. 7, was determined to be 10%. Athough this value falls within the normal range for values in material balance errors, the higher deviation compared with the value determined in the subW-H fractionation could be due to the adsorption of certain peptides on the PES membrane, as previously indicated.

**Total free amino acids.** According to the MWCO of the membranes selected in the fractionation for the Alc-H, the retention percentage of free amino acids was low with values lower than 40% for both separation steps and concentration factors close to 1 in both steps (CF_1step_ = 1.1 and CF_2nd step_ = 1.2). The total free amino acid content in the Alc-H was relatively low compared to the subW-H. Therefore, low purity indexes for total free amino acids were obtained in the R1 and R2 fractions, 3% in both streams. The purity index for total free amino acids in the P2 stream was also not high due to the relatively high protein fraction content in this stream.

The mass balance of total free amino acids was evaluated and also included in Fig. 8. 51.5% of the initial total free amino acid content was retained in the R1, 12% in R2 and 22% was determined in the final permeate P2. It must be highlighted the high percentage of the initial total free amino acids retained in R1, specially considering that MW of amino acids ranges from 75.07 Da for glycine to 204.23 for tryptophan and the MWCO of UF-Synder membrane was 10 kDa. Adsorption and interactions with the adsorbed peptides on the PES membrane surface could led to this retention of free amino acids in the R1 stream. The error of the mass balance (Eq. 7) was 14.2%. The highest value for the mass balance error was also determined for total free amino acids in the subW-H.

**Total organic carbon and nitrogen contents** were assessed in the streams of this consecutive process. The obtained values enabled the evaluation of the N:C molar ratio with similar values in the different streams: 0.30, 0.28, 0.33, 0.30 and 0.31 for F, R1, P1, R2 and P2, respectively. These values indicated that most of the organic matter correspond to protein-type compounds, according to the value reported by Rivas-Ubach (N:C = 0.3) for these compounds (Rivas-Ubach et al. 2018).

**Molecular weight distribution**. Table 2 listed the MWD of the three final streams generated (R1, R2 and P2) in the Alc-H fractionation. Slightly higher percentage of peptides with MW > 10 kDa (the MWCO of the membrane) was determined in R1 (14.2%) compared to the initial feed (11.6%), which did not show a sharp separation by the Synder-ST UF membrane. When using NP010 membrane in the second stage, NF retentate (R2) still contained peptides with MW < 2 kDa (37.1%). As it was also observed in the fractionation of the subW-H, different MW classes of peptides were determined in all streams and only peptides with a size much smaller than the MWCO of the membrane were able to permeate freely in the first UF step and the second step with the NP010 membrane.

Saidi et al. (Saidi et al. 2014a, b) also found that peptides with MW below 4 kDa were retained by a UF-4 kDa membrane in the study of fractionation of tuna dark muscle by-product hydrolysate by using Alcalase. These authors also pointed out that only small peptides passed freely through the membrane. However, these authors when passing the Alcalase hydrolysate of this by-product through the NP010 NF membrane, the NF retentate was enriched in fractions with MW > 1 kDa and < 4 kD while the NF permeate was completely free from peptide fractions with MW > 4 kDa and enriched in smaller peptide fraction, achieving a more effective separation in the NF than in the UF process.

##### Reducing and iron quelating capacity

The reducing and the iron quelating capacity of the streams generated in the separation of the Alc-H have been plotted in Fig. 7. Reducing capacity of all the streams were considerable lower compared to those of the subW-H, since the initial feed presented a low reducing capacity of 0.48 mMFe^2+^. In any case, the highest reducing capacity was observed in the R1 stream, where most of the peptide fraction was concentrated.

On the contrary, the Alc-H fractions exhibited stronger chelating capacity compared to the subW-H. The iron chelating capacity was affected by membrane fractionation since the retentates fractions presented higher capacity than the permeates one, corresponding to the peptide fractions of higher MW in the retentates fractions. Girgih et al. (Girgih et al. 2013) also reported that the increased peptide chain length led to higher iron chelating capacity on salmon protein hydrolysates. These authors proposed that the strong metal chelating properties of long-chain peptides may be due to synergistic effects of higher number of amino acid residues when compared to the shorter peptides while the lower chelating capacity of the permeates was attributed to the reduced additive and synergistic effect of the peptides in the permeate (narrow MW distribution).

## Conclusions

This paper discusses the impact of a two-step membrane fractionation process on two different hydrolysates obtained from the water soluble protein fraction from tuna fish meal generated by two different treatments, subW-CO_2_ and Alcalase^®^ treatment. These two treatments yielded two hydrolysates with different chemical composition regarding free amino acids and molecular weight of the peptides generated.

Due to the higher concentration of free amino acids and small peptides in the subW-H, this hydrolysate and its fractions could have valuable applications in pharmaceutical and cosmeceutical industries, thanks to their good solubility in a broad pH range, as determined by Barea et al. (2024). In contrast, the larger peptides obtained from the Alc-H could act as emulsions stabilizers, providing strong emulsion structure due to interfacial adsorption of large peptides in thick layers, as suggested by Barea et al. in their evaluation of the techno-functional properties of fish meal hydrolysates (Barea et al. 2024). The different properties of both hydrolysates present a great opportunity for the development of novel foods, ingredients or cosmetics.

The MWD of the two hydrolysates led to different fractionation strategies. The two step NF process applied to the subW-H resulted in a first retentate with higher protein fraction content, while maintaining its reducing capacity. In the retentate from the second NF step, 92% of FAA were retained, with a purity index of 29%, but a lower reducing capacity. This stream, rich in free amino acids, mainly alanine and glycine, could be of significant use in the food industry, as glycine is well known for its role as a sweeting agent and flavor enhancer.

On the other hand, for Alc-H a first UF and a second NF step led to a valuable first retentate with more than 65% of protein retention with a purity index of 50%, maintaining the high quelating capacity of the Alc-H and slightly higher reducing capacity.

The second step in the sequences proposed for both hydrolysates resulted in a lower membrane fouling degree, probably due to the reduced dry matter content in the stream fed to this second stage. In both processes, a relatively wide MW distribution of peptides was determined in all streams and it was concluded that these membranes did not perform a sharp separation. In any case, it is important to highlight that GPC, as a technique for determining the MWD, is based on the assumption that the elution time is an accurate predictor of MWD of the peptide mixture, which may not always be true. Additionally, in this study, the calibration curve was obtained using pullulan standard set, rather than a broad range of small peptides of different MW, which might exhibit different interactions with the GPC column.

Another limitation faced by this study was the complex and pronounced membrane fouling observed during the first fractionation step proposed for each hydrolysate, which prevented achieving the VRF of 4. To improve the results, it would be valuable to investigate strategies to reduce membrane fouling and explore novel or different membranes.

As future work, scaling-up the process for industrial applications will present a significant challenge.

## Data Availability

All data generated or analysed during this study are included in this published article.

## Abbreviations

subW: Subcritical water
FAA: Free amino acids
WSP: Water soluble protein
NF: Nanofiltration
UF: Ultrafiltration
MW: Molecular weight
subW-H: Subcritical water hydrolysate
Alc-H: Alcalase^®^ hydrolysate
MWCO: Molecular weight cut off
PES: Polyethersulfone
PE/PP: Polyethylene/propylene
PA: Polyamide
TFC: Thin-film composite
VRF: Volume reduction factor
R: Retention percentage factor
CF: Concentration factor
PI: Purification index
Y: Process yield
TC: Total carbon
IC: Inorganic carbon
TOC: Total organic carbon
TN: Total nitrogen
PCF: Propylchloroformate
FRAP: Ferric reducing antioxidant power
GCP-SEC-RID: Gel-permeation size exclusion chromatography coupled to a refraction index detector
aa: Amino acids
